# The dual roles of peptidoglycans: NOD1 and NOD2 inversely regulate bone metabolism

**DOI:** 10.1038/s12276-025-01522-0

**Published:** 2025-08-15

**Authors:** Ok-Jin Park, Jiseon Kim, Yeonjin Lim, Chaeyeon Park, Cheol-Heui Yun, Seung Hyun Han

**Affiliations:** 1 Department of Oral Microbiology and Immunology, and DRI, School of Dentistry, Seoul National University, Seoul, Republic of Korea; 2 Department of Agricultural Biotechnology, and Research Institute of Agriculture and Life Sciences, Seoul National University, Seoul, Republic of Korea; 3 Institutes of Green Bio Science and Technology, Seoul National University, Pyeongchang, Republic of Korea

**Keywords:** Bone, Differentiation, Central control of bone remodelling

## Abstract

Gut microbiota and microbial components are known to regulate bone metabolism. Peptidoglycan, a key bacterial cell wall component, is recognized by NOD1 and NOD2. Muramyl dipeptide (MDP), a ligand for NOD2 found in most bacteria, increases bone mass by promoting bone formation via Runx2 and β-catenin. However, the effects of NOD1 ligands, such as l-Ala-γ-d-glu-meso-diaminopimelic acid (TriDAP) from Gram-negative bacteria, remain poorly understood. Here, we demonstrate that, unlike MDP, TriDAP elicits bone resorption by decreasing osteoblast and Runx2 levels and increasing osteoclasts in the distal femurs of mice intraperitoneally administered TriDAP. This treatment inhibited osteoblast differentiation by downregulating Runx2 expression and also decreased the protein stability of Runx2 by increasing its ubiquitination. TriDAP, but not MDP, reduced the expression of IκB and increased NF-κB transcriptional activity in osteoblasts. The inhibition of osteoblast differentiation by TriDAP occurred through NF-κB activation and NOD1 recognition. TriDAP exhibited a marginal increase in osteoclast differentiation in the presence of RANKL, but it enhanced osteoclast differentiation in an osteoblast–osteoclast co-culture system. This suggests that TriDAP directly affects osteoblasts and indirectly affects osteoclasts. TriDAP did not induce osteoclast differentiation in the presence of NOD1-deficient osteoblasts. Other NOD1 ligands, C12-iE-DAP and C14-Tri-LAN-Gly, also inhibited osteoblastogenesis and promoted osteoclastogenesis, similar to TriDAP. *Bacillus cereus* peptidoglycan preferentially stimulates NOD1 but not NOD2, leading to increased bone resorption. In conclusion, activation of NOD1 in osteoblasts plays a role in regulating bone homeostasis by enhancing bone resorption.

## NOD1 activation disrupts bone formation and increases resorption

Bone health relies on the balance between bone formation and resorption by osteoblasts and osteoclasts, respectively. This study explores how TriDAP, often found as a part of peptidoglycan in the cell wall of bacteria, affects bone metabolism through a receptor called NOD1. Researchers used mice to study the effects of TriDAP, a NOD1 ligand, on bone health. They found that TriDAP reduced bone volume by inhibiting new bone formation and increasing bone resorption. This was linked to decreased activity of Runx2, a protein crucial for osteoblast function, and increased activity of NF-κB, a protein involved in inflammation. In conclusion, TriDAP-activated NOD1 inhibits osteoblasts while promoting osteoclasts, resulting in bone loss. This suggests that targeting NOD1 could be a potential strategy for treating bone diseases.

This summary was initially drafted using artificial intelligence, then revised and fact-checked by the author.

## Introduction

Bone undergoes continuous remodeling to maintain health, with tightly regulated processes of resorption and formation^1,2^. Osteoclasts and osteoblasts are responsible for bone resorption and bone formation, respectively. Osteoclasts are multinucleated cells (MNCs) that originate from a hematopoietic lineage. Osteoclastogenesis is differentiated by macrophage colony-stimulating factor (M-CSF) and the receptor activator of nuclear factor kappa B ligand (RANKL)^1,3^. RANKL binding to RANK triggers the differentiation of osteoclast precursors into tartrate-resistant acid phosphatase (TRAP)-positive multinucleated osteoclasts^1^. Osteoblasts originate from mesenchymal stem cells and are regulated through multiple signaling pathways that involve transforming growth factor β, bone morphogenic protein, fibroblast growth factor and Wnt/β-catenin^4^. Runt-related transcription factor 2 (Runx2) is essential for osteoblast differentiation because it induces osteogenic genes such as ALP, osteocalcin and bone sialoprotein^5^.

Recent research has shown that gut microbiota can influence bone metabolism^6^. The surface molecules of bacteria, known as microbe-associated molecular patterns, are recognized by pattern recognition receptors. This recognition leads to the induction of host immune responses^7^. Microbe-associated molecular pattern–pattern recognition receptor interactions are a central aspect of host immune activation in response to microbes. Peptidoglycan (PGN) is a major component of the cell wall in most bacteria^8^. The dry weight of Gram-positive bacteria is approximately 90%, whereas that of Gram-negative bacteria is about 10%^9^. PGN is a large polymeric molecule consisting of linear glycan chains composed of two amino sugars, *N*-acetylglucosamine (GlcNAc) and *N*-acetylmuramic acid. The short peptide, consisting of three to five amino acids, binds to GlcNAc^8^. Gram-positive bacteria predominantly possess lysine-type PGN, whereas Gram-negative bacteria are characterized by diaminopimelic acid (DAP)-type PGN^10^. In mammals, polymeric PGN is digested by human PGN recognition protein-2, lysozymes and *N*-acetyl-d-glucosaminidase^11,12^, with the predominant PGN fragment being *N*-acetylmuramic acid tripeptide^13^. PGN fragments derived from intestinal microbiota have been identified in the bone marrow^14^.

The cytosolic receptors nucleotide-binding oligomerization domain (NOD) 1 and NOD2 recognize distinct bacterial PGN-derived motifs, γ-d-glutamyl-meso-diaminopimelic acid (iE-DAP) and muramyl dipeptide (MDP), respectively. Their activation elicits pro-inflammatory responses through nuclear factor kappa B (NF-κB) signaling, which can contribute to the onset and progression of inflammatory diseases^15^. However, NOD1 and NOD2 signaling have been reported to trigger opposing host immune responses. For instance, the NOD1 ligand, but not the NOD2 ligand, induces pro-inflammatory cytokines and chemokines in the adipose tissue of patients with gestational diabetes^16^. Meanwhile, PGN derived from *Lactobacillus salivarius* Ls33 and M-Tri-Lys, another NOD2 ligand, shows an anti-inflammatory capacity in a murine trinitrobenzene sulfonic acid colitis model^17^. We previously reported that MDP, a representative NOD2 ligand, induces new bone formation^18^ and alleviates the osteoporosis caused by estrogen-deficiency^19^. MDP enhances osteoblast activity by activating Runx2 and the canonical Wnt–β-catenin pathway via Wnt3a induction^19^. In addition, it decreases osteoclast activity by downregulating the RANKL/osteoprotegerin (OPG) ratio^18,19^. As NOD1 and NOD2 activations induce distinct immune responses, they could differentially regulate bone metabolism. While the role of NOD2 signaling in promoting bone mass has been well established, the involvement of NOD1 signaling in bone metabolism remains poorly characterized. Therefore, in this study, we investigated the effects of NOD1 activation on bone metabolism using a representative NOD1 ligand, l-Ala-γ-d-glu-meso-DAP (TriDAP), and NOD1-activating PGN.

## Materials and methods

### Materials

TriDAP, C12-iE-DAP, C14-Tri-LAN-Gly, MDP and Pam3CSK4 were obtained from InvivoGen. Fetal bovine serum (FBS) was purchased from Gibco. α-Minimum Essential Medium (α-MEM), ascorbic acid–free α-MEM and Dulbecco’s modified Eagle medium (DMEM) were purchased from Welgene. Trypsin-ethylenediaminetetraacetic acid (EDTA), phosphate-buffered saline (PBS) and penicillin–streptomycin were purchased from Hyclone. Murine soluble RANKL and mouse M-CSF were obtained from R&D Systems and PeproTech, respectively. 1α,25-Dihydroxyvitamin D3 was purchased from EMD Millipore. Ascorbic acid, β-glycerophosphate, *N*-*p*-tosyl-l-phenylalanine chloromethyl ketone (TPCK), cycloheximide (CHX) and mutanolysine were obtained from Sigma-Aldrich Chemical Inc. An alkaline phosphatase (ALP) staining kit and alizarin red S solution were purchased from EMD Millipore. A TRAP staining kit was purchased from Cosmo Bio Co. Primary antibodies specific to p65, IκBα and p-NF-κB were purchased from Cell Signaling Technology. Antibodies to Myc epitope tag and hemaglutinin (HA) epitope tag were purchased from Abcam and InvivoGen, respectively. The antibody to β-actin was obtained from Santa Cruz Biotechnology. Goat anti-rabbit IgG-horseradish peroxidase (HRP) and goat-anti-mouse IgG-HRP were purchased from Southern Biotech. Tryptic soy broth and de Man, Rogosa, and Sharpe medium were purchased from BD Biosciences.

### Purification of *Bacillus cereus* PGN (Bc.PGN)

*B. cereus* KCTC 13153 was obtained from the Korean Collection for Type Culture. *B. cereus* was grown at 37 °C to the mid-log phase in aerobic conditions. The bacterial pellet was collected by centrifugation, washed with PBS three times and stored at -80 °C until use. PGN was isolated as previously described with minor modifications^20^. Soluble PGN was prepared by incubating 100 μg of insoluble PGN with 50 U of mutanolysin at 37 °C with shaking for 24 h. The enzyme was inactivated by incubation at 100 °C for 10 min.

### Mice

All animal experiments were approved by the Institutional Animal Care and Use Committee of Seoul National University (approval no. SNU-160524-3). Five-week-old C57BL/6 mice and 1-day-old neonatal mice were purchased from Orient Bio. NOD1-deficient mice were kindly provided by Professor Jong-Hwan Park (Chonnam National University, Gwangju, Republic of Korea).

### Micro-computed tomography (micro-CT)

Mice that had acclimated for 1 week were randomly divided into three groups and intraperitoneally administered 100 μl of 1.25 mg/kg TriDAP (*n* = 5), 100 μl of 1.25 mg/kg MDP (*n* = 5) or 100 μl of PBS on days 0 and 4. On day 7, the mice were euthanized, and their femurs were removed and fixed in 10% formalin. In a separate experiment, 6-week-old male C57BL/6 mice were randomly divided into two groups and treated with PBS or insoluble Bc.PGN (30 μg) via oral gavage three times per week for 4 weeks. Their femurs were scanned using micro-CT (Skyscan1275, Bruker-CT, Kontich, Belgium) and quantitatively analyzed as described previously^18^.

### Histological analysis

The femurs were fixed and decalcified in 10% EDTA in PBS for 7 days at 4 °C. The decalcified femurs were paraffin embedded and sectioned and then subjected to hematoxylin and eosin (H&E) or TRAP staining. Images were obtained using a BX-51 fluorescence microscope equipped with a DP72 camera (BX-51, Olympus). TRAP-positive areas were measured using ImageJ software (National Institutes of Health).

### In vivo calcein double labeling

Mice were intraperitoneally treated with 20 mg/kg of calcein one day before the initial TriDAP administration and after the final TriDAP administration. TriDAP (1.25 mg/kg) or PBS was administered to the mice on days 1 and 5. On day 9 after the first calcein injection, the mice were euthanized, and their femurs were fixed and embedded in methyl methacrylate. The resin blocks were longitudinally sectioned, and the calcein-labeled bone tissue was examined using a BX-51 fluorescence microscope equipped with a DP72 camera (Olympus). The mineral apposition rate was measured using OsteoMeasure software (OsteoMetrics).

### Osteoblast differentiation

Mouse osteoblast precursors were isolated from the calvaria of 1-day-old C57BL/6 mice, due to their incomplete ossification and the high abundance of osteoprogenitor cells, as previously described^18,21^. In brief, the calvariae were digested in α-MEM containing 1% penicillin–streptomycin, 0.1% collagenase (Wako) and 0.2% dispase (Roche) for 15 min at 37 °C with shaking. The digestion procedure was repeated five times, and the supernatant was collected in a single tube, except for the first collection. The cells (5 × 10^5^) were plated onto a 100-mm dish and incubated for 3 days. In a separate experiment, MC3T3-E1 cells, a well-characterized murine osteoblast precursor cell line^22^, were used for various in vitro assays, including intracellular signaling and transfection with reporter genes.

### Osteoclast differentiation

Bone marrow cells isolated from the femurs and tibiae of 5-week-old mice with fully developed bone marrow were incubated in α-MEM supplemented with 10% FBS, 100 U/ml penicillin and 100 μg/ml streptomycin in the presence of 5 ng/ml M-CSF for 1 day. Nonadherent cells were obtained and induced to differentiate into bone marrow-derived macrophages (BMMs) by incubation with 20 ng/ml M-CSF for 4 days. The BMMs were plated onto a 96-well culture plate at 2 × 10^4^ cells/well and incubated with 20 ng/ml RANKL and 20 ng/ml M-CSF in the presence or absence of TriDAP, C12-iE-DAP, C14-Tri-LAN-Gly or soluble Bc.PGN. In co-culture experiments, BMMs (1 × 10^5^) and calvarial osteoblast precursors (1 × 10^4^ cells per well) plated onto a 48-well culture plate were incubated with 10 mM β-glycerophosphate, 50 μg/ml ascorbic acid and 100 ng/ml 1α,25-dihydroxyvitamin D_3_ in the presence or absence of soluble Bc.PGN for 12 days. After that incubation, the cells were fixed and stained using a TRAP staining kit according to the manufacturer’s recommendation. We used an inverted phase-contrast microscope to enumerate TRAP-positive MNCs with three or more nuclei as osteoclasts.

### Reverse-transcriptase polymerase chain reaction (RT-PCR)

Total RNA was extracted and subjected to RT-PCR or real-time RT-PCR as described previously^23^. The primer sequences were as follows: *Alp*: forward 5′-CCAACTCTTTTGTGCCAGAGA-3′ and reverse 5′-GGCTACATTGGTGTTGAGCTTTT-3′; *Bone sialoprotein* (*Bsp*) forward 5′-GAATGTGTGTCCTCTGAAG-3′ and reverse 5′-AATCCTCGCTCTCTGCATGG-3′; *Runx2* forward 5′-AACGATCTGAGATTTGTGGGC-3′ and reverse 5′-CCT GCGTGGGATTTCTTGGTT-3′; *Glyceraldehyde 3-phosphate dehydrogenase* (*Gapdh*) forward 5′-AGGTCGGTGTGAACCGGATTTG-3′ and reverse 5′-TGTAGACCATGTAGTTGAGGTCA-3′.

### Western blotting

HEK293 cells were transiently transfected with the Myc-tagged Runx2 and/or Smurf1 expression constructs using Attractene transfection reagent (Qiagen) as recommended by the manufacturer. The transfected cells were treated with 40 μg/ml CHX in the presence or absence of 1 μg/ml TriDAP for 2, 4 or 8 h. In a separate experiment, primary calvarial osteoblasts were treated with 1 μg/ml TriDAP for 15, 30, 60 or 90 min. The cells were lysed and subjected to western blotting as previously described^24^. In a separate experiment to examine Runx2 ubiquitination, HEK293 cells were co-transfected with MYC-tagged Runx2 and HA-tagged ubiquitin. After 24 h, the cells were lysed, and proteins were immunoprecipitated as previously described^24^.

### Immunofluorescence staining

MC3T3-E1 cells were treated with 1 µg/ml TriDAP for 15 min. The cells were fixed and sequentially stained with an antibody specific to p65 and Hoechst 33342, as previously described^18^.

### Enzyme-linked immunosorbent assay (ELISA)

The OPG and RANKL levels were measured using Quantikine ELISA kits (R&D Systems) according to the manufacturer’s instructions.

### Reporter gene assay

HEK293 cells (2 × 10^4^ cells/0.2 ml), seeded onto a 96-well culture plate in DMEM, were incubated overnight. The cells were transfected with pNF-κB-Luc and pRL-TK *Renilla* luciferase plasmids (Promega), together with the plasmid encoding human NOD1 or NOD2, using LipofectamineTM2000 transfection reagent (Invitrogen) for 3 h in serum-starved DMEM. After the medium was replaced with fresh DMEM containing 10% FBS, 100 U/ml penicillin and 100 μg/ml streptomycin, the cells were incubated for a further 24 h and then stimulated with PGN for 16 h. The cells were lysed, and the firefly and *Renilla* luciferase activities were analyzed using a dual luciferase reporter assay system (Promega) in accordance with the manufacturer’s instructions.

### Statistical analysis

All experiments were performed at least three times. All quantitative results from in vivo studies and in vitro studies are expressed as mean values ± standard error of the mean and mean values ± standard deviation, respectively. The statistical differences were determined using the unpaired *t*-test or one-way analysis of variance. An asterisk indicates a statistically significant difference from the control group at *P* < 0.05.

## Results

### TriDAP decreases bone volume by downregulating new bone formation

**Fig. 1:**
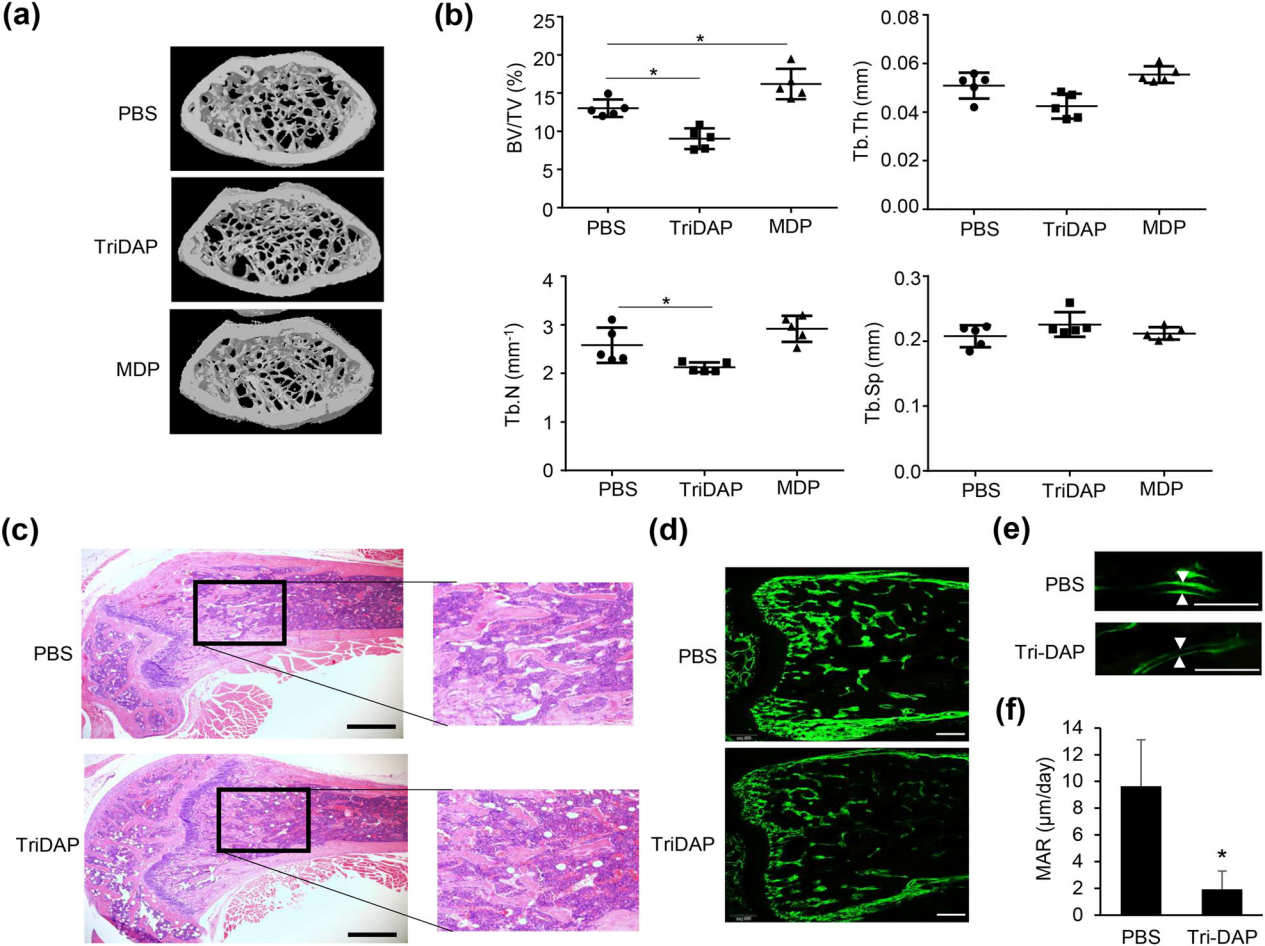
TriDAP but not MDP decreases bone mass. Mice were intraperitoneally administered 1.25 mg/kg of TriDAP or MDP, or PBS, on days 0 and 4. **a** On day 7, micro-CT images were obtained of the distal femurs of those mice. **b** BV/TV, Tb.Th, Tb.N and Tb.Sp. were analyzed using a CT analyzer. BV/TV, trabecular bone volume; Tb.Th, trabecular thickness; Tb.N, trabecular number; Tb.Sp., trabecular separation. **c** Distal femur sections from mice treated with PBS or TriDAP were stained with H&E and photographed with an inverted phase-contrast microscope. **d** Mice were intraperitoneally administered calcein one day before the first TriDAP administration and after the second TriDAP administration. Resin blocks of those femurs were sectioned. The images were obtained using a fluorescent microscope. **e** The distance between two labels represents the mineral apposition rate (MAR). Scale bars, 500 μm. **f** The MAR was quantified using OsteoMeasure. *P < 0.05.

To examine the effects of TriDAP on bone metabolism, mice were intraperitoneally treated with TriDAP, MDP or PBS. Administration with TriDAP, in contrast to MDP, resulted in a significant reduction in trabecular bone volume, trabecular thickness and trabecular number compared with the control mice (Fig. 1a, b), although trabecular separation remained unchanged. Femur sections stained with H&E show the decreased trabecular bone in TriDAP-treated mice (Fig. 1c). To determine whether TriDAP affects new bone formation, we conducted a calcein labeling assay. As shown in Fig. 1d, the calcein double-labeling images indicate a reduction in the calcein-labeled mineralized surface of the distal femur in mice treated with TriDAP compared with control mice. The administration of TriDAP to mice resulted in a significant decrease in the mineral apposition rate, suggesting inhibition of bone formation (Fig. 1e, f). These results demonstrate that TriDAP inhibits bone mass by downregulating new bone formation.

### TriDAP attenuates osteogenesis via downregulation of Runx2 activity

**Fig. 2:**
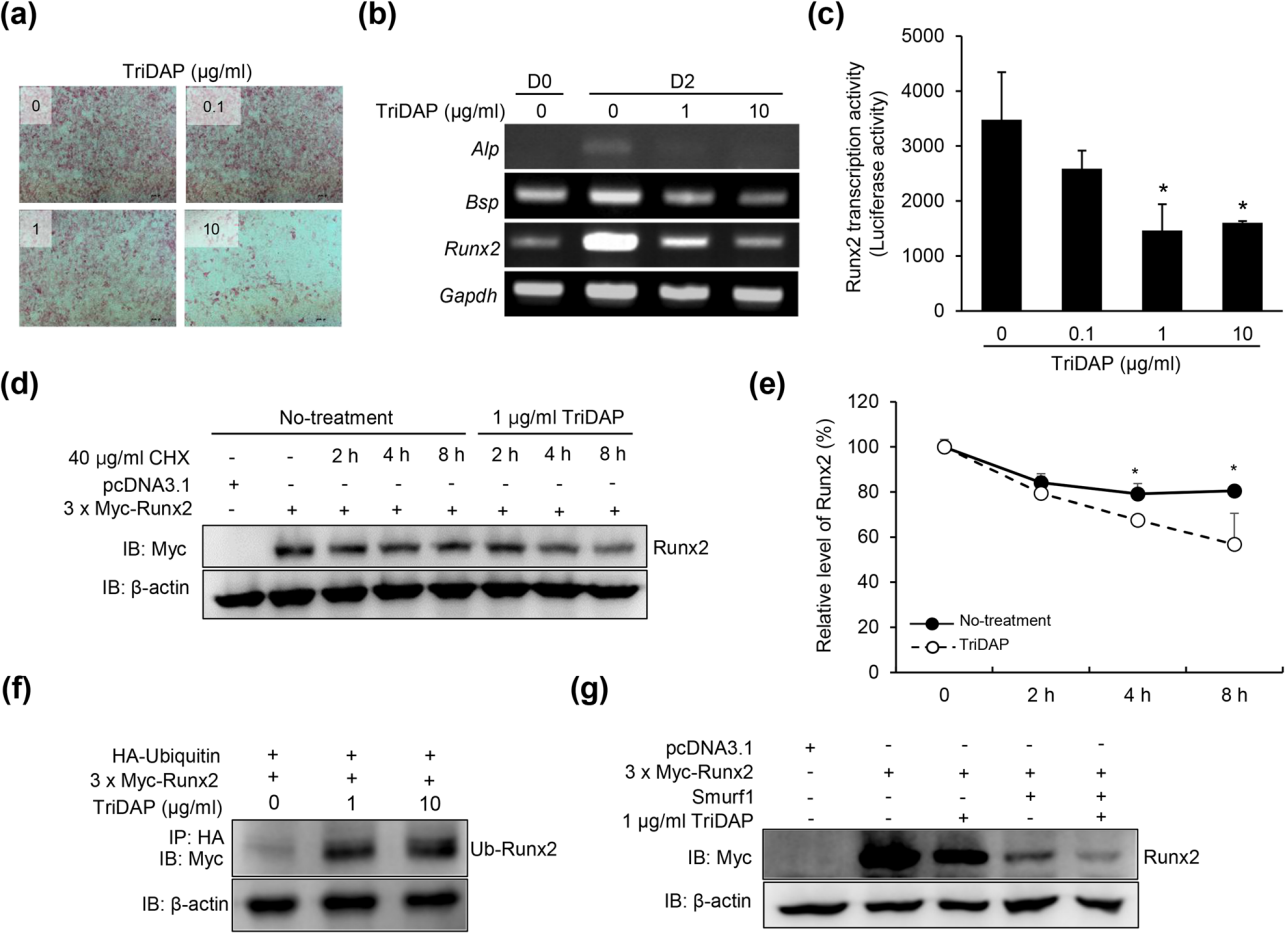
TriDAP inhibits osteoblast differentiation by decreasing Runx2 activity. **a** Calvarial osteoblast precursors were differentiated for 6 days with 50 μg/ml ascorbic acid and 10 mM β-glycerophosphate, with or without TriDAP, and then subjected to ALP staining. **b** MC3T3-E1 cells were treated for 2 days with 50 μg/ml ascorbic acid and 10 mM β-glycerophosphate, with or without TriDAP. The expression levels of *Alp*, *Bsp*, *Runx2* and *Gapdh* were determined using RT-PCR. **c** MC3T3-E1 cells were transfected with 6× OSE-Luc plasmids. The cells were treated with 0, 0.1, 1 or 10 μg/ml TriDAP for 18 h, lysed and subjected to a luciferase assay. **d** HEK293 cells were transiently transfected with 3×Myc-Runx2; treated with CHX (40 µg/ml) in the presence of TriDAP (1 µg/ml) for 2, 4 or 8 h; and then lysed. The expression of Runx2 was determined by western blotting using a specific anti-Myc antibody. **e** The relative expression level of Runx2 was normalized to that of β-actin. **f** HEK293 cells were co-transfected with 3×Myc-Runx2 and HA-ubiquitin and then treated with TriDAP for 24 h. The cells were lysed, and immunoprecipitation was performed using an anti-HA antibody, followed by immunoblotting with a anti-Myc antibody. **g** HEK293 cells were co-transfected with 3×Myc-Runx2 and Smurf1 expression vectors, treated with TriDAP for 24 h and lysed. The Runx2 level was determined by western blotting with a specific anti-Myc antibody. *P < 0.05.

As shown in Fig. 1, we observed that TriDAP inhibits new bone formation. Therefore, we examined whether TriDAP inhibits osteoblast differentiation. Primary calvarial osteoblast precursors were differentiated in the presence or absence of TriDAP and then subjected to ALP staining. As expected, TriDAP inhibited osteoblast differentiation in a dose-dependent manner (Fig. 2a). The expression of osteogenic marker genes such as *Alp*, *Bsp* and *Runx2* was dose-dependently inhibited by TriDAP (Fig. 2b). Runx2 serves as a master transcription factor in osteoblast differentiation^25^, and TriDAP inhibited Runx2 transcriptional activity (Fig. 2c). We further examined whether TriDAP regulates Runx2 protein stability. The half-life of the Runx2 protein was decreased in cells treated with TriDAP (Fig. 2d, e). Runx2 undergoes degradation through a ubiquitin–proteasome-dependent pathway^26^. The degradation of Runx2 is mediated by Smurf1, an E3 ubiquitin ligase^26^. TriDAP increased the ubiquitination of Runx2 (Fig. 2f), and the Runx2 protein was more efficiently decreased by TriDAP in the presence of Smurf1 than in its absence (Fig. 2g). These results suggest that TriDAP inhibits osteoblast differentiation by downregulating Runx2 stability.

### TriDAP primarily induces osteoclastogenesis by upregulating the RANKL/OPG ratio

**Fig. 3:**
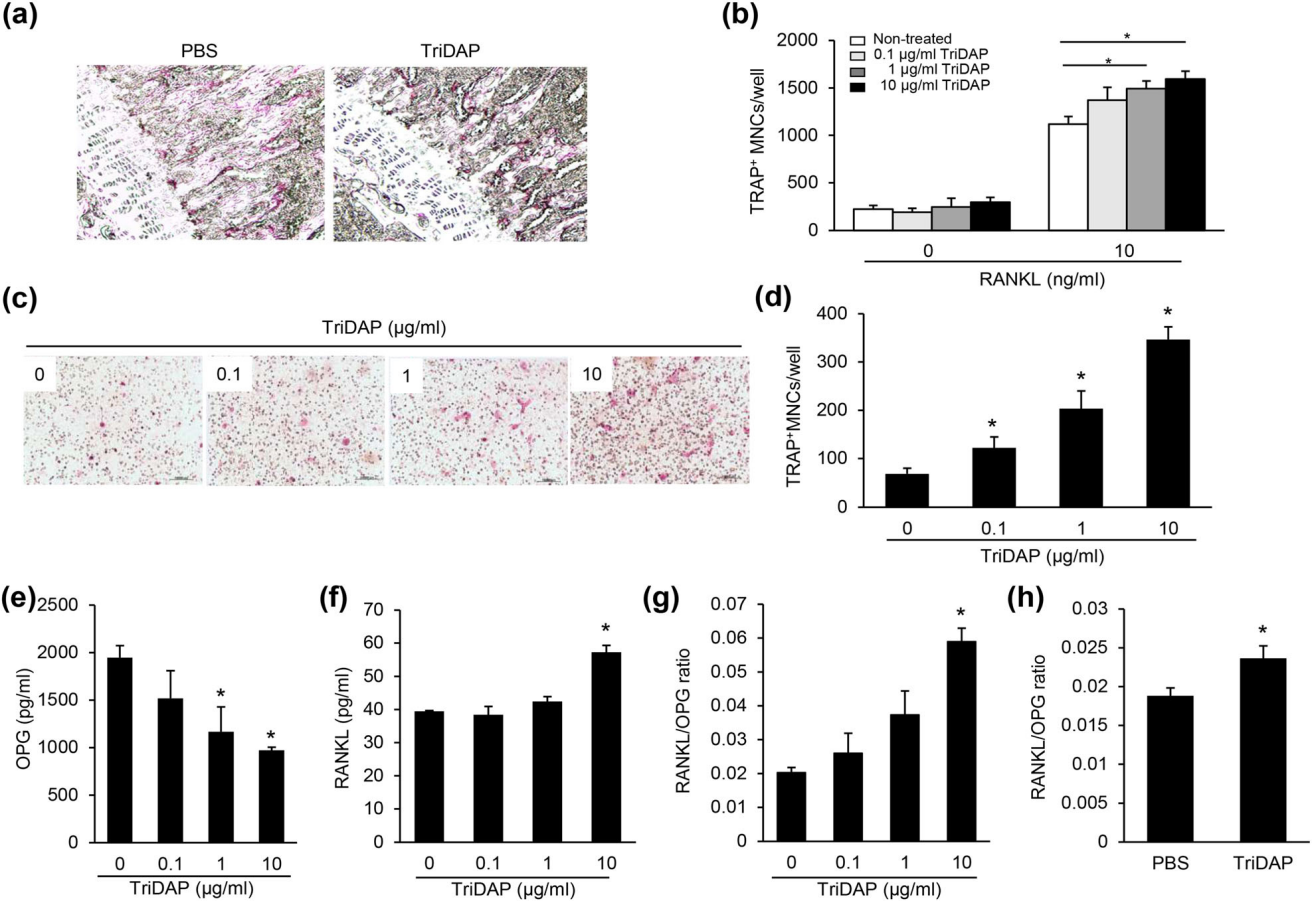
TriDAP indirectly enhances osteoclast differentiation by increasing the RANKL/OPG ratio in osteoblasts. **a** The femurs of mice treated with PBS or TriDAP were subjected to TRAP staining to determine osteoclast activity. Asterisks denote TRAP-positive osteoclasts on the bone surface. **b** BMMs were treated with RANKL and M-CSF for 3 days. The RANKL-primed cells were treated with M-CSF and TriDAP with or without additional RANKL for one day and then fixed and stained with TRAP. TRAP^+^ MNCs with three or more nuclei were enumerated using an inverted microscope. **c**, **d** BMMs were co-cultured with calvarial osteoblast precursors. The cells were differentiated with 50 μg/ml ascorbic acid and 10 mM β-glycerophosphate supplemented with 10 nM 1α,25-dihydroxyvitamine (OH)_2_D_3_ in the presence or absence of TriDAP. On day 6, the cells were fixed and stained with TRAP (**c**). TRAP-positive MNCs with three or more nuclei were enumerated using an inverted microscope (**d**). **e**–**g** On day 2, culture supernatants were collected to measure the expression of OPG (**e**) and RANKL (**f**) by ELISA and the RANKL/OPG ratio (**g**) is shown. **h** Mice were intraperitoneally treated with 1.25 mg/kg of TriDAP or PBS. After 18 h, bone marrow extracellular fluids were collected. The levels of OPG and RANKL were measured using ELISA. *P < 0.05.

We subsequently examined whether the negative effect of TriDAP on bone metabolism is due to enhanced osteoclast activity. Figure 3a shows that the TRAP-positive surface area in femur sections subjected to TRAP staining was increased in TriDAP-treated mice compared with control mice, indicating that TriDAP increases osteoclast activity in vivo. Next, an in vitro osteoclast differentiation assay was performed to clarify the effect of TriDAP on osteoclast differentiation. In the absence of RANKL, TriDAP failed to induce osteoclast differentiation (Fig. 3b). In the presence of RANKL, the synthetic bacterial lipoprotein PAM3CSK4 significantly increased the number of TRAP-positive MNCs differentiated from RANKL-primed BMMs, whereas TriDAP alone produced only a modest increase (Fig. 3b and Supplementary Fig. 1). TriDAP, however, did produce an increase in TRAP-positive MNCs in the osteoblast–osteoclast co-culture system (Fig. 3c, d). TriDAP induced a decrease in OPG expression and an increase in RANKL expression, increasing the RANKL/OPG ratio (Fig. 3e–g). Our in vivo observations also showed an increased RANKL/OPG ratio (Fig. 3h). These results indicate that TriDAP predominantly enhances osteoclast differentiation by upregulating the RANKL/OPG ratio.

### NF-κB activation is important for TriDAP responsiveness

**Fig. 4:**
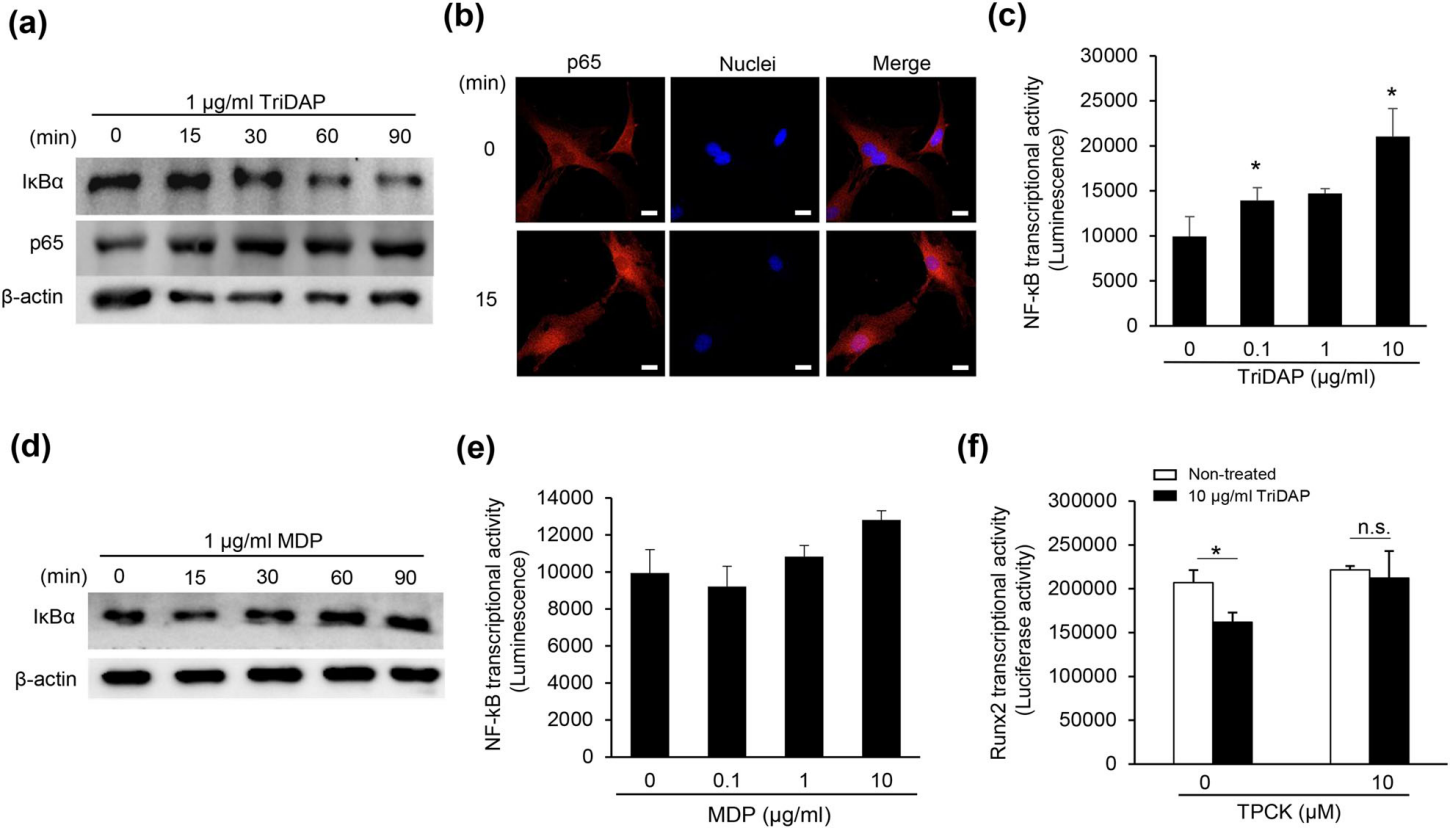
NF-κB activation is a critical factor in TriDAP-inhibited osteoblast differentiation. **a** MC3T3-E1 cells were treated with 1 µg/ml TriDAP for 15, 30, 60 or 90 min. The cells were lysed and subjected to western blotting using specific antibodies to IκBα or p65. **b** MC3T3-E1 cells were treated with 1 µg/ml TriDAP for 15 min and then fixed and stained sequentially with specific antibodies to p65 and Hoechst 33342. Cell images were obtained using a confocal microscope. Scale bar, 20 μm. **c**, **e** MC3T3-E1 cells were transfected with an NF-κB-Luc plasmid and treated with 0, 0.1, 1 or 10 µg/ml TriDAP (**c**) or MDP (**e**) for 24 h. The cells were lysed and subjected to a luciferase assay. **d** MC3T3-E1 cells were treated with 1 µg/ml of MDP for 15, 30, 60 or 90 min. The cells were lysed and subjected to western blotting using specific antibodies to IκBα. **f** MC3T3-E1 cells were transfected with 6× OSE-Luc plasmids. The cells were pretreated with 10 μM TPCK for 1 h, followed by stimulation with 10 μg/ml TriDAP for an additional 23 h. The cells were lysed and subjected to a luciferase assay. *P < 0.05. n.s. not significant.

NF-κB activation is known to inhibit osteoblast differentiation^27^. Conversely, NOD1 receptor ligands induce the activation of NF-κB^28^. Thus, we examined NF-κB activation in osteoblasts treated with TriDAP, which decreased IκBα expression (Fig. 4a) but increased the expression and nuclear translocation of p65 (Fig. 4a, b). TriDAP increased NF-κB transcriptional activity in a dose-dependent manner (Fig. 4c). However, MDP did not affect IκBα expression or NF-κB transcriptional activity (Fig. 4d, e). These results indicate that, unlike MDP (a representative NOD2 ligand), TriDAP activates NF-κB in osteoblasts. When the cells were pretreated with TPCK, an NF-κB inhibitor, the inhibitory effect of TriDAP on Runx2 transcriptional activity was significanly reduced (Fig. 4f). These results suggest that NF-κB activation is critical for the inhibition of osteoblast differentiation by TriDAP.

### Nod1 is critical to TriDAP-induced bone resorption

**Fig. 5:**
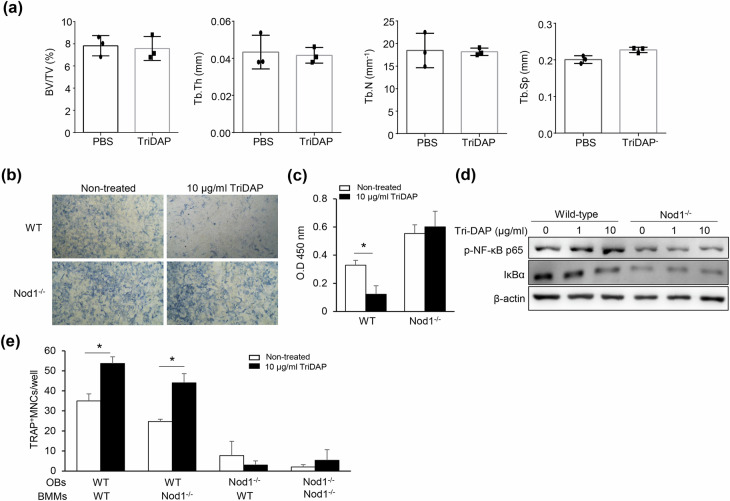
NOD1 is essential for TriDAP effects on bone metabolism. NOD1-deficient mice were intraperitoneally treated with 1.25 mg/kg of TriDAP or PBS on days 0 and 4. On day 7, the distal femurs were obtained from the mice. **a** Each femur was scanned by micro-CT, and the trabecular bone parameters were measured using Skyscan programs. Three-dimensional images of each femur were obtained using CT volume software. **b** BV/TV, Tb.N., Tb.Sp. and Tb.Th. were calculated using the CT analyzer. **c** Calvarial osteoblast precursors from wild-type (WT) or NOD1-deficent mice were differentiated for 10 days with 50 μg/ml ascorbic acid and 10 mM β-glycerophosphate with or without TriDAP. The cells were fixed and subjected to alizarin red S staining. **d** Calvarial osteoblast precursors from WT or NOD1-deficent mice were treated with 1 µg/ml TriDAP for 15 min and then lysed and subjected to western blotting using specific antibodies to p-IκBα or p65. **e** WT or NOD1-deficient BMMs were co-cultured with WT or NOD1-deficient calvarial osteoblast precursors. The cells were differentiated with 50 μg/ml ascorbic acid and 10 mM β-glycerophosphate supplemented with 10 nM 1α,25(OH)_2_D_3_ in the presence or absence of TriDAP. On day 6, the cells were fixed and stained with TRAP. TRAP-positive MNCs with three or more nuclei were enumerated using an inverted microscope.

TriDAP is recognized by NOD1 and then induces inflammatory responses through NF-κB activation^29^. Thus, we investigated whether NOD1 is required for TriDAP responsiveness using NOD1-knockout mice and NOD1-deficient osteoblasts. When NOD1-knockout mice were intraperitoneally treated with TriDAP, the structural trabecular bone parameters did not differ from those of PBS-treated NOD1-knockout mice (Fig. 5a). The TriDAP-decreased ALP staining and osteoblast mineralization were not observed in NOD1-deficient osteoblasts, suggesting that NOD1 plays an essential role in the inhibition of osteoblast differentiation caused by TriDAP (Fig. 5b, c). TriDAP did not lead to the phosphorylation of NF-κB p65 nor did it decrease IκB expression in NOD1-deficient osteoblasts, indicating that NF-κB activation by TriDAP did not occur in those cells (Fig. 5d). Furthermore, in NOD1-deficient osteoblasts, TriDAP failed to induce the differentiation of both wild-type and NOD1-deficient macrophages into osteoclasts (Fig. 5e), suggesting that osteoblasts, not osteoclasts, are the target cells of TriDAP. Collectively, these results indicate that TriDAP recognition by NOD1 is essential for its effects on bone metabolism.

### Nod1 ligands inhibit osteogenesis and increase RANKL-mediated osteoclastogenesis

**Fig. 6:**
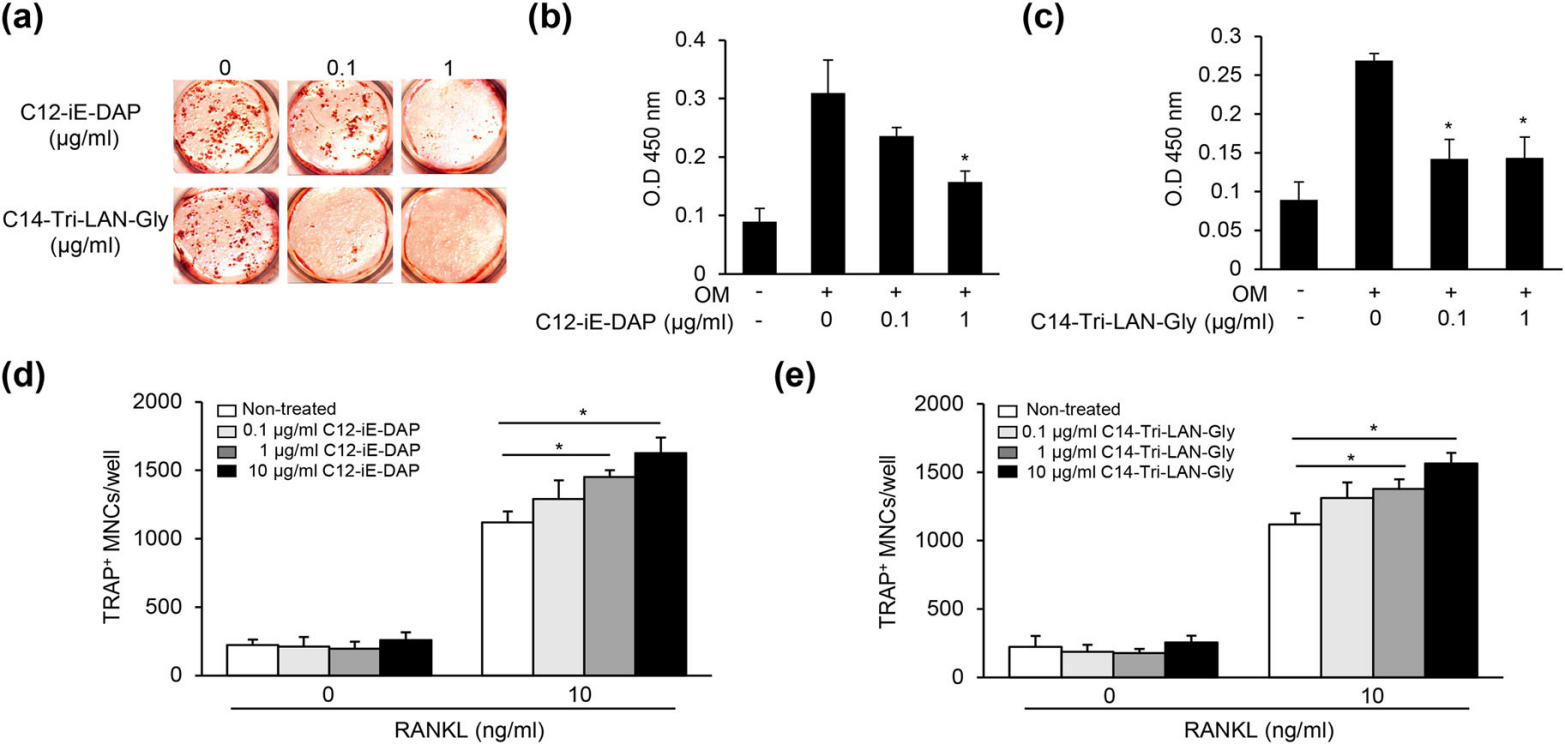
NOD1 agonists inhibit osteoblast differentiation and increase osteoclast differentiation. **a**–**c** Calvarial osteoblast precursors were treated with C12-iE-DAP or C14-Tri-LAN-Gly in the presence of 50 μg/ml ascorbic acid and 10 mM β-glycerophosphate for 14 days. The cells were fixed and subjected to alizarin red S staining (**a**). Alizarin red S precipitates were dissolved and measured by a spectrophotometric analysis at 450 nm (**b**, **c**). **d**, **e** BMMs were treated with RANKL and M-CSF for 3 days. The RANKL-primed cells were treated with C12-iE-DAP (**d**) or C14-Tri-LAN-Gly (**e**) in the presence or absence of additional RANKL for 1 day. The cells were fixed and stained with TRAP. TRAP-positive MNCs with three or more nuclei were enumerated using an inverted microscope.

Next, we investigated whether the inhibition of osteogenesis and increase of osteoclastogenesis are general phenomena associated with NOD1 ligands. Alizarin red S staining showed that the NOD1 ligands C12-iE-DAP and C14-Tri-LAN-Gly effectively inhibit calcium deposition, suggesting the general attenuation of osteoblast differentiation by NOD1 ligands (Fig. 6a–c). NOD1 ligands also increased the number of TRAP-positive MNCs that differentiated from RANKL-primed BMMs in the presence of RANKL (Fig. 6d, e). Collectively, NOD1 ligands lead to the inhibition of osteogenesis and enhancement of osteoclastogenesis.

### NOD1-activating Bc.PGN induces bone destruction

**Fig. 7:**
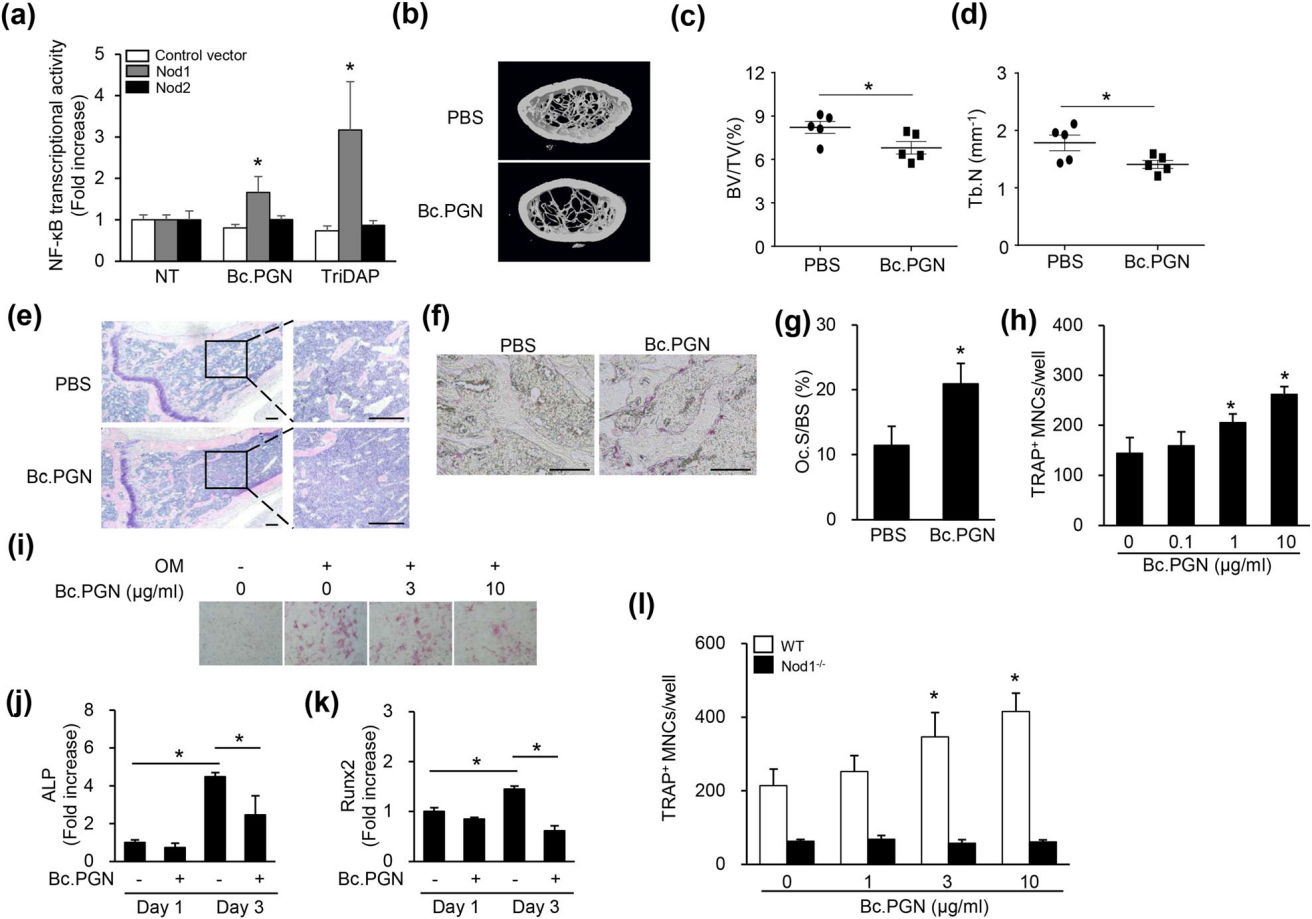
*Bacillus cereus* PGN preferentially stimulates NOD1 and increases bone resorption. **a** HEK293 cells were transiently transfected with a plasmid expressing human NOD1 or NOD2 in the presence of the firefly luciferase reporter plasmid regulated by an NF-κB transcription factor and the pRL-TK *Renilla* luciferase plasmid. The cells were incubated with or without soluble Bc.PGN or TriDAP at 10 μg/ml for 16 h; lysed; and measured for firefly or *Renilla* luciferase activity. Firefly luciferase activity was normalized to *Renilla* luciferase activity. **b**–**g** Seven-week-old male mice (*n* = 5) received intragastric administrations of PBS or insoluble Bc.PGN (30 μg) three times weekly for 4 weeks. Their femurs were scanned by micro-CT, and trabecular bone parameters were measured using Skyscan. Three-dimensional images of the femurs were obtained using CT volume software (**b**). BV/TV (**c**) and Tb.N. (**d**) were calculated using the CT analyzer. Paraffin sections of the decalcified femurs were subjected to H&E and photographed (**e**). Scale bars, 200 μm. Paraffin sections of the decalcified femurs were subjected to TRAP staining and photographed (**f**). The Oc.S/BS ratio was measured using Osteomeasure software (**g**). Scale bars, 200 μm. Oc.S/BS, osteoclast surface per bone surface. **h** RANKL-primed BMMs were differentiated into osteoclasts with M-CSF in the presence of soluble Bc.PGN at 0, 0.1, 1 or 10 μg/ml for 2 days. The cells were then fixed and subjected to TRAP staining. TRAP-positive MNCs with three or more nuclei were enumerated. **i** Calvarial osteoblast precursors were differentiated into osteoblasts by incubation with β-glycerophosphate and ascorbic acid in the presence of soluble Bc.PGN at 0, 3 or 10 μg/ml for 12 days. The cells were then fixed and subjected to ALP staining. **j**, **k** Calvarial osteoblast precursors were stimulated with β-glycerophosphate and ascorbic acid in the presence or absence of soluble Bc.PGN (3 μg/ml) for 1 or 3 day(s). Total RNA was isolated, and the mRNA expression levels of ALP (**j**) and Runx2 (**k**) with GAPDH were determined by real-time RT-PCR. **l** Wild-type or NOD1-deficient RANKL-primed cells were stimulated with Bc.PGN (0, 1, 3 or 10 μg/ml) in the presence of M-CSF/RANKL for 2 days. The cells were then fixed and stained with TRAP. TRAP-positive MNCs with three or more nuclei were enumerated using an inverted microscope. OM, osteogenic medium containing 10  mM β-glycerophosphate and 50  μg/ml ascorbic acid. *P < 0.05.

Given that NOD1 signaling activation induces bone destruction, we examined whether a PGN that preferentially stimulates NOD1 also induces bone destruction. Bc.PGN and TriDAP enhanced NF-κB transcriptional activity in NOD1-transfected cells, but they showed minimal effects in NOD2-transfected cells, indicating that Bc.PGN selectively activates NOD1 signaling (Fig. 7a). Bc.PGN-treated mice exhibited decreases in trabecular bone volume and trabecular bone number relative to control mice (Fig. 7b–d). However, trabecular separation and trabecular thickness were unchanged by Bc.PGN (data not shown). Femur sections from Bc.PGN-treated mice showed reduced bone area (Fig. 7e) and increased bone surface (Fig. 7f, g). Bc.PGN produced a dose-dependent increase in osteoclast differentiation in vitro (Fig. 7h), and it attenuated osteoblast differentiation (Fig. 7i). The expression of osteogenic marker genes, such as ALP and Runx2, was significantly decreased by Bc.PGN (Fig. 7j, k). In addition, Bc.PGN did not induce osteoclastogenesis in NOD1-deficient osteoblasts (Fig. 7l). Therefore, NOD1 activation by Bc.PGN increases bone resorption through the induction of osteoclast activity and the suppression of osteoblast activity.

## Discussion

PGNs could be recognized by NOD1 and/or NOD2, which have subtle differences in their molecular structures^30^. Generally, the activation of NOD1 and NOD2 induces pro-inflammatory responses through NF-κB activation^15^. In this study, we report that NOD1 and NOD2 signaling pathways appear to have opposing effects on the regulation of bone metabolism. Previous reports indicated that NOD2 ligands increase bone mass by inducing osteoblast activity and reducing osteoclast activity^18,19^. By contrast, NOD1 ligands enhanced bone resorption by downregulating new bone formation. As shown in Supplementary Fig. 2, TriDAP enhanced bone resorption by downregulating both osteoblast differentiation and the stability of Runx2. In contrast to MDP, TriDAP also increased NF-κB activation in osteoblasts. NOD1 seems to be critical to the effects of TriDAP on bone metabolism, directly affecting osteoblasts while having no effect on osteoclasts. Therefore, NOD1 signaling, in contrast to NOD2 signaling, might regulate bone homeostasis by inducing bone resorption.

This study demonstrated that, in contrast to NOD2, NOD1 activation by synthetic ligands or a NOD1-activating PGN induces bone resorption. In alignment with our findings, Jiao et al. reported that NOD1 signaling plays an important role in the development of periodontitis, characterized by the destruction of alveolar bone^31^. NOD1-deficient mice exhibited reduced bone resorption compared with wild-type mice. An accumulation of NOD1-activating bacteria, specifically NI1060, was observed at the disease lesion, along with alveolar bone resorption caused by NI1060. NI1060 has been proposed to contribute to the development of periodontitis through alveolar bone resorption by inducing neutrophil recruitment and pro-inflammatory mediators. Although it remains unconfirmed whether *Actinomyces naeslundii* PGN activates NOD1, it has been demonstrated that *A. naeslundii* PGN induces alveolar bone resorption via osteoclastogenesis^32^. Our findings suggest that *A. naeslundii* PGN might activate NOD1. We propose that a NOD1 signaling blockade could serve as a therapeutic target for controlling inflammatory bone disease.

The indirect regulation of osteoclast differentiation by triggering osteoblasts is thought to be a common phenomenon in NOD1 and NOD2 signaling. In this study, TriDAP alone did not induce the differentiation of RANKL-primed BMMs. TriDAP resulted in minimal osteoclast differentiation in the presence of RANKL; however, it efficiently promoted osteoclastogenesis by upregulating the RANKL/OPG ratio in an osteoblast and osteoclast co-culture system. Furthermore, co-culturing NOD1-deficient osteoblasts with wild-type BMMs did not result in osteoclast differentiation. However, as expected, a co-culture of wild-type osteoblasts and NOD1-deficient osteoclasts induced osteoclast differentiation. A previous report indicated that the NOD2 ligand MDP affects bone homeostasis primarily through direct actions on osteoblasts rather than osteoclasts^18^. Unlike TriDAP, bacterial cell wall components such as synthetic lipoproteins and lipopolysaccharide efficiently induce osteoclastogenesis independent of RANKL^33,34^. NOD1 is ubiquitously expressed in various cell types, whereas NOD2 is expressed only in specific cells, including macrophages, osteoblasts and intestinal cells^30^. Interestingly, although NOD1 is also present in macrophages as precursors of osteoclasts, its ligands predominantly affect osteoblasts rather than osteoclasts. Further research is needed to examine how cell tropism influences bone metabolism through PGN.

The downregulation of Runx2, mediated by the NOD1–NF-κB axis, contributes to the inhibition of osteoblast differentiation. TriDAP increased Runx2 degradation by upregulating its ubiquitination, and Smurf1 enhanced the TriDAP-inhibition of Runx2. Previous studies indicate that both the canonical and noncanonical NF-κB signaling pathways inhibit bone formation by downregulating osteoblast differentiation^35–37^. NF-κB activation has been shown to decrease new bone formation by reducing the interaction of Runx2 and β-catenin with the osteocalcin/bone sialoprotein promoter^38^. Tumor necrosis factor alpha attenuates Runx2 expression by increasing Runx2 ubiquitination and upregulating Smurf1 expression^39^. Takami et al. reported that a small anti-NF-κB peptide derived from nuclear acidic protein alleviated ovariectomy-induced bone loss^40^. This anti-NF-κB peptide increased osteoblastogenesis by inducing Smad1 phosphorylation and decreased osteoclastogenesis by inhibiting NF-κB transcriptional activity. Thus, we propose that targeting the NOD1–NF-κB–Runx2 axis in osteoblasts could serve as a potential therapeutic strategy for bone loss.

NOD1 and NOD2 signaling pathways are both recognized for their roles in NF-κB activation^29^, but they have contrasting effects on bone metabolism. In previous reports, we demonstrated that MDP, a NOD2 ligand, induces bone formation by upregulating Runx2 and alleviates bone loss in OVX mice by inducing canonical WnT–β-catenin signaling^18,19^. In the present study, NOD1 ligands induced bone resorption and concurrently downregulated bone formation. Specifically, NOD1 ligands, in contrast to NOD2 ligands, inhibit Runx2 expression and enhance NF-κB activation in osteoblasts. MDP activates canonical Wnt–β-catenin signaling^19^. However, because Wnt–β-catenin signaling is well known to play a role in upregulating bone formation, we hypothesize that TriDAP might inhibit canonical Wnt–β-catenin signaling. Consistent with our findings, the NOD1 ligand, but not the NOD2 ligand, induces inflammatory responses in adipose tissue^16^. Fernandez et al. reported that oral administration of whole bacteria and PGN from *L. salivarius* Ls33 alleviated colitis in a NOD2-dependent manner^17^. NOD2 ligands have been shown to reduce the expression of the pro-inflammatory cytokines IL-1β and CXCL2 while increasing IL-10 expression, suggesting that they have anti-inflammatory properties. NOD2, in contrast to NOD1, activates antiviral responses in cells infected with human respiratory syncytial virus by inducing IRF3 and IFN-β production^41^. Taken together, these findings indicate that NOD1 and NOD2 have opposing effects on bone metabolism. Further research is required to identify the specific factors that contribute to this discrepancy.

Dysbiosis of the gut microbiota has been observed in OVX mice and patients with osteoporosis^42,43^. Factors such as aging and antibiotic use are known to alter gut microbiota composition^44,45^. During bacterial growth, PGN is constantly released, and antibiotic administration can trigger a PGN storm^46^. Notably, NOD1 activation by PGN derived from the gut microbiota has been shown to enhance innate immunity^14^. This study has demonstrated that, in contrast to NOD2, NOD1 synthetic ligands and PGN induce bone resorption. These findings are particularly important for elucidating the mechanisms underlying inflammatory bone diseases triggered by bacterial infections or microbiome abnormalities, and for identifying potential therapeutic targets in conditions associated with excessive osteoblast activity. Proper bone homeostasis, maintained by bone-forming osteoblasts and bone-resorbing osteoclasts, is essential for skeletal health. Therefore, NOD1 signaling may represent a novel therapeutic target for treating bone diseases such as osteopetrosis, melorheostosis and heterotopic ossification.

## Supplementary information

Supplementary Information
