# Supplementary material for: The dual roles of peptidoglycans: NOD1 and NOD2 inversely regulate bone metabolism
Source: Exp Mol Med. 2025 Aug 15;57(8):1837–46. doi: 10.1038/s12276-025-01522-0 (PMC12411613; doi:10.1038/s12276-025-01522-0)
Supplement: Supplementary file 1 — Supplementary Information [file 12276_2025_1522_MOESM1_ESM.pdf]

Supplementary materials

The dual roles of peptidoglycans: NOD1 and NOD2 inversely regulate bone metabolism

Ok-Jin Park[1], Jiseon Kim[1], Yeonjin Lim[1], Cheol-Heui Yun[2,3], Seung Hyun Han[1, *]

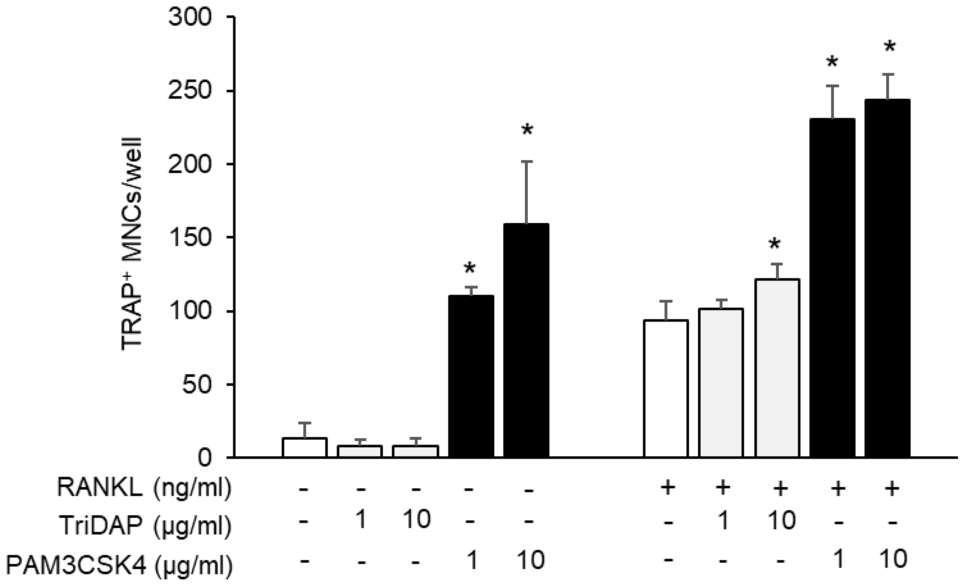

Supplementary Fig. 1. Unlike PAM3CSK4, TriDAP increases osteoclast differentiation at extremely low levels, even in the presence of RANKL. RANKL-primed cells were treated with TriDAP or PAM3CSK4 at 1 or 10 µg/ml with or without additional RANKL for one day. The cells were fixed and stained with TRAP. TRAP-positive MNCs with three or more nuclei were enumerated using an inverted microscope. *$P < 0.05$.

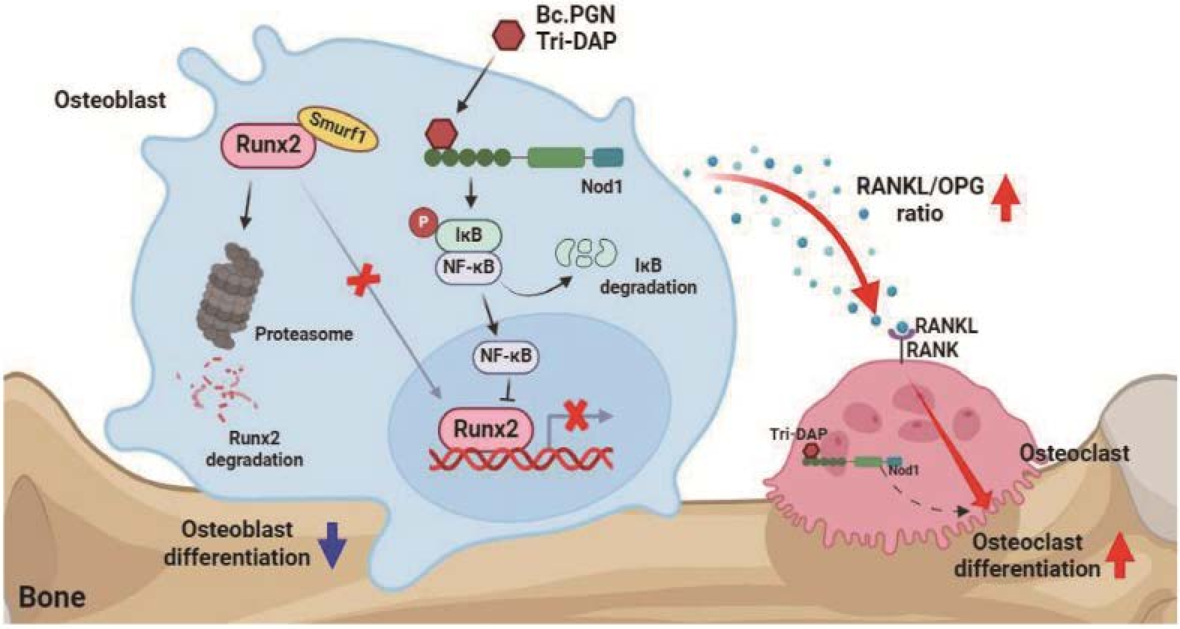

Supplementary Fig. 2. Proposed molecular mechanisms for TriDAP-inhibited osteoclast differentiation.
